# Identifying complications and failure modes of innovative growing rod configurations using the (hybrid) magnetically controlled growing rod (MCGR) and the spring distraction system (SDS)

**DOI:** 10.1007/s43390-021-00378-4

**Published:** 2021-06-22

**Authors:** Justin V. C. Lemans, Casper S. Tabeling, René M. Castelein, Moyo C. Kruyt

**Affiliations:** Department of Orthopaedic Surgery, University Medical Center Utrecht, PO Box 85500, 3508 GA Utrecht, The Netherlands

**Keywords:** Early-onset scoliosis, Growth-friendly, Magnetically controlled growing rod, Spring distraction system, Complications, Implant failure

## Abstract

**Background:**

Magnetically controlled growing rods (MCGRs) offer non-invasive distractions in Early-Onset Scoliosis (EOS). However, implant-related complications are common, reducing its cost-effectiveness. To improve MCGRs functionality and cost-effectiveness, we often combine a single MCGR with a contralateral sliding rod (hybrid MCGR). Recently, we developed the spring distraction system (SDS) as an alternative, which provides continuous distraction forces through a helical spring. This study aims to identify complication rates and failure modes of EOS patients treated with either of these innovative systems.

**Methods:**

This single-centre retrospective study included EOS patients treated with a (hybrid) MCGR or SDS between 2013 and 2018. Baseline demographics, and data regarding complications and implant growth were measured. Complication rate, complication profile, complication-free survival and implant growth were compared between groups.

**Results:**

Eleven hybrid- and three bilateral MCGR patients (4.1-year follow-up) and one unilateral, eleven hybrid and six bilateral SDS patients (3.0-year follow-up) were included. Groups had similar age, sex, aetiology distribution, and pre-operative Cobb angle. Complication rate was 0.35 complications/patient/year for MCGR patients and 0.33 complications/patient/year for SDS patients. The most common complications were failure to distract (MCGR-group; 8/20 complications) and implant prominence (SDS-group; 5/18 complications). Median complication-free survival was 2.6 years, with no differences between groups (*p* = 0.673). Implant growth was significantly higher in the SDS-group (10.1 mm/year), compared to the MCGR-group (6.3 mm/year).

**Conclusion:**

(Hybrid) MCGR and SDS patients have similar complication rates and complication-free survival. Complication profile differs between the groups, with frequent failure to distract leading to significantly reduced implant growth in (hybrid) MCGR patients, whereas SDS patients frequently exhibit implant prominence and implant kyphosis.

**Level of evidence:**

III.

**Supplementary Information:**

The online version contains supplementary material available at 10.1007/s43390-021-00378-4.

## Introduction

Early onset scoliosis (EOS), if left untreated, is a life-threatening condition [1]. The challenge in surgical EOS treatment is to control the curve while maintaining adequate spinal growth. Traditional Growing Rod (TGR) treatment, wherein rods are periodically surgically distracted, is associated with high rates of wound complications and increased anaesthetic exposure, with potential adverse neurodevelopmental effects [2]. In contrast, the Magnetically Controlled Growing Rod (MCGR) offers non-invasive distractions, thus removing the need for re-operations [3]. However, frequent lengthening procedures are still required. In addition, the MCGR is difficult to contour, and implant-related complications are frequent, with an incidence of almost 50% during the first 2–3 years [4]. Many of these complications are mechanical in nature, like anchor failures, rod fractures and a failure to distract [5]. This last category includes specific failure modes of the internal mechanism (e.g. drive pin or lead screw fractures), and is hypothesised to be caused by high-frictional forces inside the actuator [6, 7]. While newer versions of the MCGR have been developed, mechanical complications remain prevalent [8]. The re-operations necessary to correct these complications are a serious burden for the patient and increases treatment cost dramatically, potentially making MCGR treatment less cost-effective than previously described, as calculations were based only on a relatively short follow-up [9–12]. To improve MCGRs cost-effectiveness, and to provide apical control, we often combined one MCGR on the curve concavity with a contralateral rod fixated to the apex which can slide freely proximally and distally. Several studies have shown that this innovative hybrid configuration shows similar results compared to bilateral MCGR use [13, 14]. However, even in the hybrid configuration, some MCGR disadvantages remain, such as the difficulty contouring the MCGR rod, and the necessity of repetitive lengthenings.

**Fig. 1:**
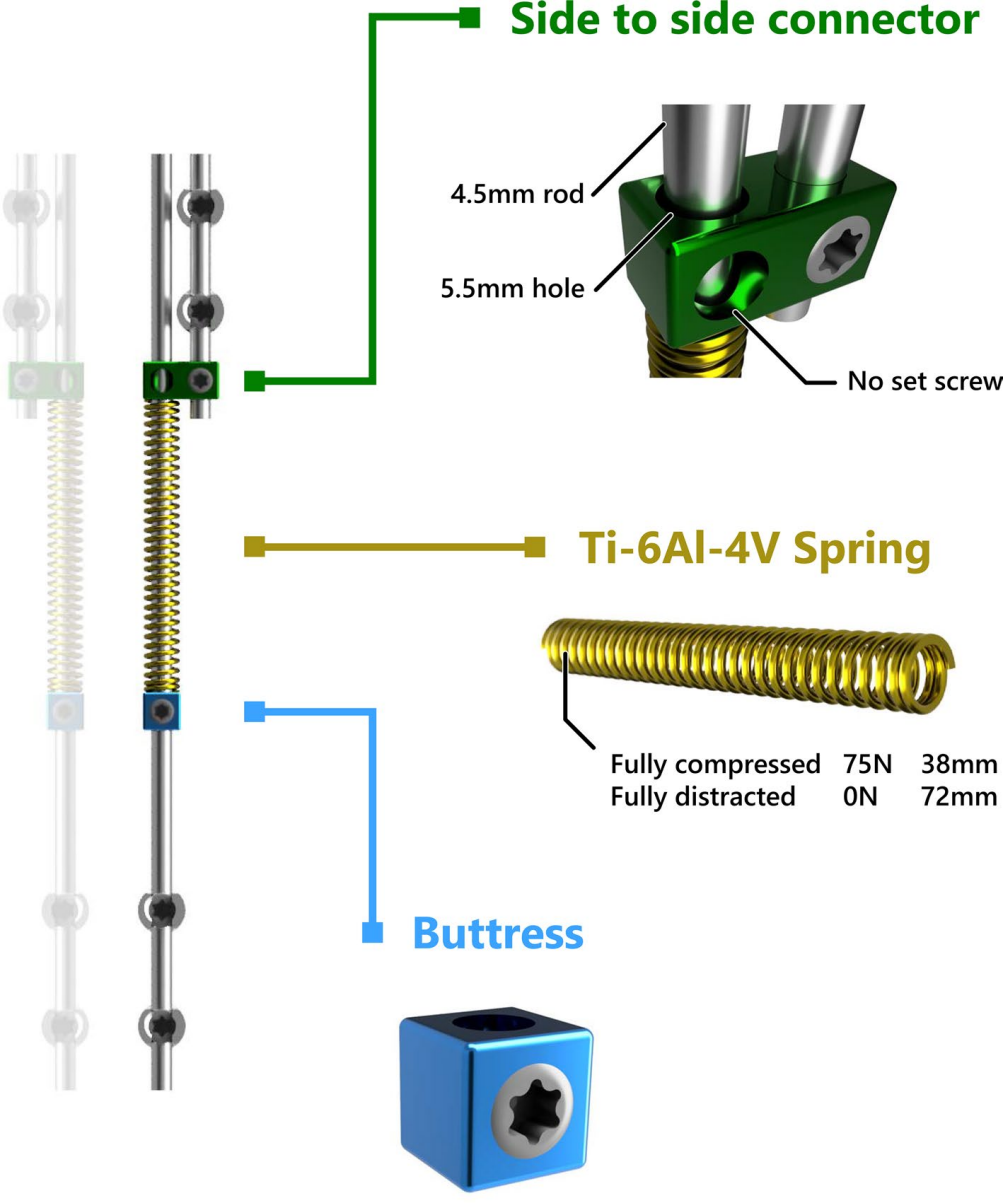
Spring distraction system concept. The SDS consists of three parts that are added to a traditional screw-rod construct: (1) The side-to-side connector (green) with one oversized hole through which a rod can slide freely. (2) The spring (gold) can be compressed over the rod by (3) the buttress (blue) during surgery, and then provides a continuous distraction force

Recently, we developed the spring distraction system (SDS), which is based on a continuous distraction aided growth-guidance concept. This system exerts a continuous distractive force with a compressed titanium spring that is positioned around a sliding rod (Fig. 1). This implant has important advantages, such as the potential to further reduce the curve after insertion and the fact that it does not have to be periodically lengthened. The design of the SDS and its preliminary and 2-year follow-up clinical performance have recently been reported [15, 16]. However, its provisional design is not yet fully optimised, as the connectors are used off-label and do not prevent the release of metal debris. To ultimately improve these innovative growing-rod constructs in terms of complications and failure modes, understanding of the specific strengths and weaknesses of both systems is essential. Therefore, the current study aims to report and compare follow-up adjusted complication rate and complication profile of EOS patients treated with either the (hybrid) MCGR or SDS. Secondary aims are to describe complication-free survival, and implant growth.

## Materials and methods

### Study design and study period

This study was approved by the Institutional Review Board of the UMC Utrecht [METC 18/638 and METC 16/276]. Data were collected from all EOS patients implanted with either a unilateral, bilateral- or hybrid MCGR or SDS between 2013 and 2018. Our institution used MCGR exclusively from the end of 2013 until October 2016. Since then, patients have the option to participate in a prospective clinical study investigating the SDS (*G*rowing *R*ods with the *A*ddition of a *D*istraction *S*pring—GRADS study). Before study approval, an extensive Investigational Medical Device Dossier including risk analysis was created in accordance with the European Medical Device Regulations (MDR). All patients before October 2016 received the MCGR, while most (18/19) eligible patients after this date opted for the SDS. Patients that were revised from another growing-rod system to either MCGR or SDS were excluded.

### Surgical techniques

**Fig. 2:**
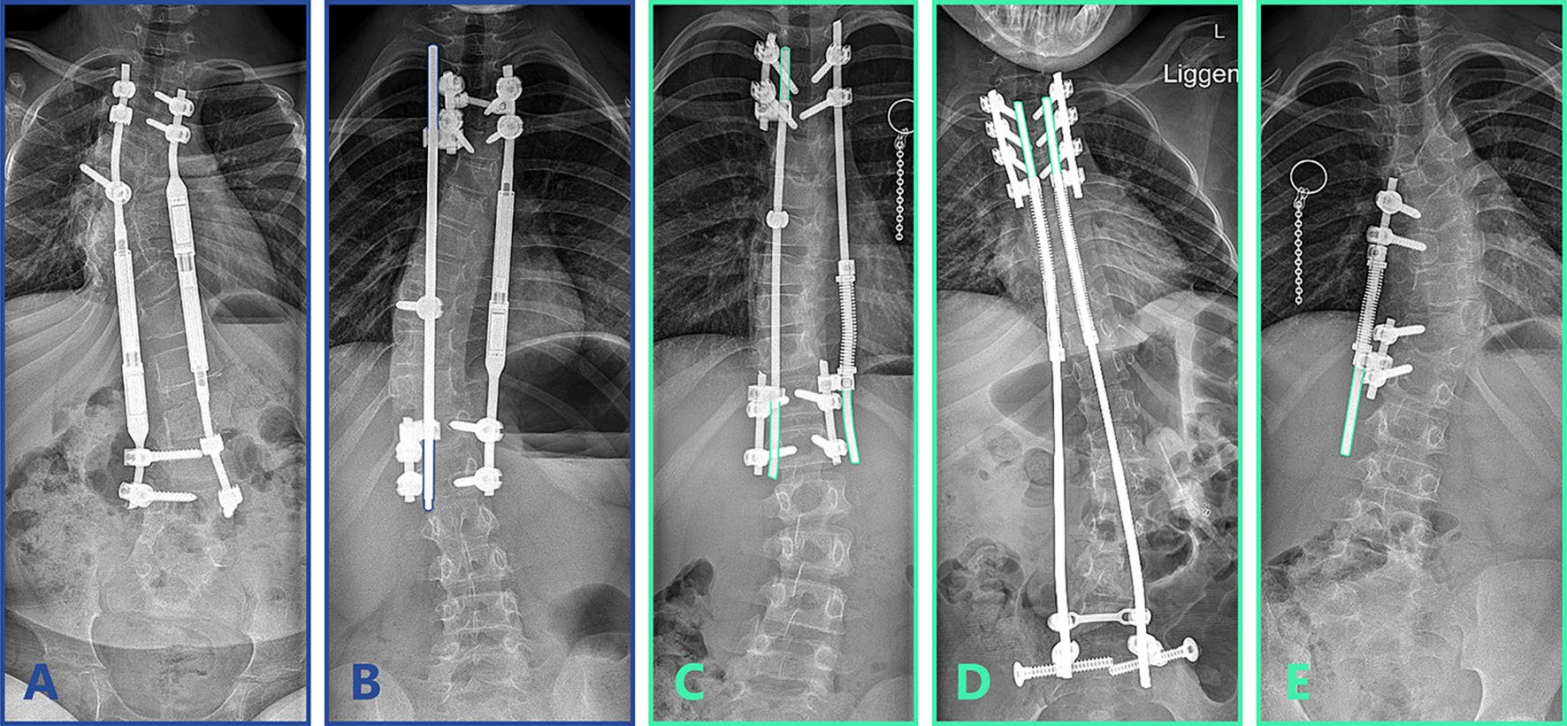
Implant configurations. Different MCGR and SDS configurations, coloured rod outlines represent the parts of the rod that can freely slide. **a** Bilateral (offset) MCGR**. b** Unilateral concave MCGR combined with a convex sliding rod that is fixated to the apex for apical control (hybrid). The convex rod can freely slide through the proximal and distal side-to-side connectors. **c** Unilateral concave SDS combined with a convex sliding rod that is fixated to the apex for apical control. The convex rod can freely slide through the proximal and distal side-to-side connectors. **d** Bilateral SDS fixated to the pelvis with ilio-sacral screws. **e** Unilateral concave SDS without a convex rod

All patients underwent intra-operative neuromonitoring. For both implant systems, anchors on at least two subsequent levels were placed proximally and distally, to which the 4.5 or 5.5 mm growth-friendly constructs were mounted. In neuromuscular patients with a main curve that extended to the pelvis, bilateral iliosacral screws (Tanit, EUROS, La Ciotat, France) were used distally. Three MCGR patients received a bilateral MCGR (MAGEC, NuVasive, San Diego, CA, USA) (Fig. 2a), the other 11 MCGR patients received a hybrid construct, with an MCGR on the curve concavity and a sliding rod fixed to the apical level on the curve convexity (Fig. 2b), as previously described [13, 14]. MCGR patients were lengthened at the outpatient clinic once every 2–3 months, where distraction was performed in a prone position until the Electronic Remote Control showed ≥ 10 mm or clunking was felt. In case of failure-to-distract, a new lengthening was attempted after 3 months. If that failed, a trial was done under anaesthesia with manual traction. If all these failed and the curve progressed, this was a reason to revise the implant. For the SDS, in idiopathic and syndromic patients, a similar hybrid configuration with a concave SDS and convex sliding rod was used (Fig. 2c). In neuromuscular and most congenital patients, a bilateral SDS was implanted (Fig. 2d). One congenital SDS patient received only a concave SDS, with no contralateral rod (Fig. 2e). At the end of surgery, intrawound vancomycin was left in the deep and superficial wound. Drains were not routinely used. No post-operative braces were used, and there were no restrictions in activities after surgery.

### Data collection

Demographic-, disease-specific- and surgical parameters as well as data regarding implant-related complications were obtained through review of the electronic patient record and the spinal radiographs. Complication type, interval between initial surgery and onset of complications and the necessity for re-operation was recorded, irrespective of whether the re-operation had already taken place or was postponed to be treated with final fusion surgery. When an implant had reached its maximum distraction length (4–6 cm) and had to be replaced (MCGR) or re-tensioned (SDS), this was not deemed a complication, but the re-operation was counted towards the total number of re-operations. Specific complications that were evaluated and their diagnostic criteria are shown in Table 1.

**Table 1:** Evaluated complications

Complication	Definition used
Neurological injury	Neurological deficit that is either permanent or that necessitates a re-operation. This does not include temporary loss of neuromonitoring signals
Anchor complications	Screws or hooks that loosen or exhibit pull-out or cut-out
Rod complications	Rod fractures or rod slippage
Failure to distract	No radiological implant growth during two consecutive MCGR lengthenings (MCGR) or during 6-months follow-up (SDS) May be caused by: 1. MCGR driving rod/actuator failure 2. SDS spring/connector/buttress failure 3. Spontaneous fusion
Proximal junctional kyphosis	Angle between PIV and PIV + 2 ≥ 10°, and increase ≥ 10° compared to pre-operatively [34]
Implant prominence	Prominence of the implant through the soft tissues, causing local pain or skin breakdown
Wound dehiscence	Loss of integrity of the closed surgical wound
Superficial SSI	CDC criteria for superficial SSI [35]
Deep SSI	CDC criteria for deep SSI [35]
Late SSI	Conforming to CDC criteria for SSI except for time of occurrence (30- and 90 days for superficial- and deep SSI’s, respectively) [35]
Other	Any complication necessitating a re-operation not mentioned above

*MCGR* magnetically controlled growing rod, *SDS* spring distraction system, *SSI* surgical site infection, *CDC* Centres for Disease Control and Prevention, *PIV* proximal instrumented vertebra

In addition, during each outpatient clinic visit in which a spinal radiography was performed (generally every 6 months), cumulative length increase in the MCGR actuator or SDS spring was measured and plotted over time. Measurements were performed on calibrated radiographs and were normalised for coronal and sagittal tilt of the actuator or spring. All chart reviews and radiographic measurements were performed independently by two observers (JVCL and CST). Disagreements were discussed until consensus was reached. Radiographic length measurements were averaged between both observers. A two-way mixed intraclass correlation coefficient of 0.993 showed that there was excellent correlation between the observers.

### Statistical analysis

Summaries of demographic and radiographic data were reported as mean with standard deviation (SD). Baseline characteristics were compared between groups with a Chi-squared test (categorical data) or independent *t*-test (continuous data). The number of complications per patient was calculated and normalised for the mean follow-up length to find the number of complications/patient/year.

The complication data were also used to perform a Kaplan–Meier survival analysis comparing both groups. The outcome was the occurrence of a complication and survival time was thus the time until the first complication occurred. Patients who did not suffer a complication were censored at their latest follow-up date. The survival curves of both groups were statistically compared with the Log-Rank test. Depending on whether the proportional hazard assumption was met, the hazard ratio was used to compare the instantaneous risk of complications between both groups.

To compare implant growth between groups, implant length at latest follow-up was used to calculate the linear annual growth rate with a linear regression analysis. Implant length at the first post-operative erect radiograph (*t* = 0) was set at 0. As the cumulative implant length increase was compared to this value, an intercept-free regression was performed. The slope of both groups was then compared with an independent t-test. Statistical analyses were performed with IBM SPSS Statistics 25.0.0.2 (IBM Corp., Armonk, NY, USA). The Kaplan–Meier survival analyses and regression analyses were performed with GraphPad Prism 9.0.0 (GraphPad Software, San Diego, CA, USA). Two-tailed statistical significance was set at *p* < 0.05.

## Results

### Population characteristics

In total, 14 MCGR (11 hybrid and 3 bilateral constructs) and 18 SDS patients (one unilateral, 11 hybrid and six bilateral constructs) were consecutively included. Patient characteristics are summarised in Table 2. Mean age at surgery was 7.9 ± 1.6 and 8.4 ± 1.9 years for the MCGR and SDS group, respectively. Mean follow-up was 4.1 ± 1.6 years for the MCGR group and 3.0 ± 0.4 years for the SDS group (*p* = 0.025). Surgery time and time to discharge were similar between both groups. Pre- and post-operative Cobb angles were similar in both groups, the MCGR group showed a post-operative correction of 44%, for SDS this was 48%. Cobb angle at latest follow-up was higher in the MCGR group, 53.5° vs. 39.8° (*p* = 0.029). A higher proportion of SDS patients received fixation to the pelvis, compared to the MCGR group (SDS: 7/18, MCGR: 1/14; *p* = 0.040), and all SDS patients received a 4.5 mm system while most patients in the MCGR group (10/14) received a 5.5 mm system (*p* < 0.001).

**Table 2:** Patient demographics

	MCGR (*N* = 14)	SDS (*N* = 18)	*p* value
Male	5/14	10/18	0.266
Age at surgery (years)	7.9 ± 1.6	8.4 ± 1.9	0.436
EOS aetiology			0.585
Idiopathic	4	3	
Congenital	3	4	
Syndromic	3	2	
Neuromuscular	4	9	
BMI (kg/m^2^)^a^	16.8 ± 3.1	15.9 ± 2.6	0.380
Surgery time (minutes)^a^	203 ± 73	221 ± 51	0.418
Time to discharge (days)^a^	7.8 ± 3.4	8.3 ± 10.0	0.845
Follow-up (years)	4.1 ± 1.6	3.0 ± 0.4	0.025
Cobb angle (°)			
Pre-operatively	70.3 ± 20.9	66.2 ± 13.6	0.507
Post-operatively	39.6 ± 19.5	34.3 ± 13.0	0.364
Latest follow-up	53.5 ± 18.6	39.8 ± 15.1	0.029
Pelvic fixation^a^	1/14	7/18	0.040
Implant configuration^a^			0.467
Unilateral concave distraction only	0	1	
Unilateral concave distraction + convex sliding rod	11	11	
Bilateral distraction	3	6	
Rod diameter^a^			< 0.001
4.5 mm	4	18	
5.5 mm	10	0	

*EOS* Early Onset Scoliosis, *BMI* Body Mass Index

^a^Measurements associated with the initial surgery

### Complication rate

Overall, implant- and procedure-related complications were common in both groups (Table 3). In the (hybrid) MCGR group, there were 20 (1.4/patient), which corresponded to 0.35 complications/patient/year. Ten MCGR patients (71%) suffered from at least one such complication. In the SDS group, 18 (1.0/patient) complications were observed, corresponding to a similar rate of 0.33 complications/patient/year. Eleven SDS patients (61%) showed at least one complication.

**Table 3:** Incidence of implant- or procedure-related complications

	MCGR		SDS	
Neurological injury	1		0	
Anchor complications	4		3	
Proximal anchor		2		0
Apical anchor		1		0
Distal anchor		1		3
Rod complications	2		4	
Rod fracture		2		3
Rod slippage		0		1
Failure to distract	8		2	
MCGR actuator failure		8		0
Side-to-side connector failure		0		2
Rod growing out of connector due to fast growth	0		1	
Proximal junctional kyphosis	3		0	
Implant prominence	0		5	
Wound dehiscence	0		1	
Superficial SSI	1		0	
Deep SSI	0		1	
Late superficial SSI	1		1	
Total number of complications	20		18	
Complications per patient	1.4		1.0	
Complications per patient per year	0.35		0.33	

*SSI* surgical site infection

### Complication profile

**Fig. 3:**
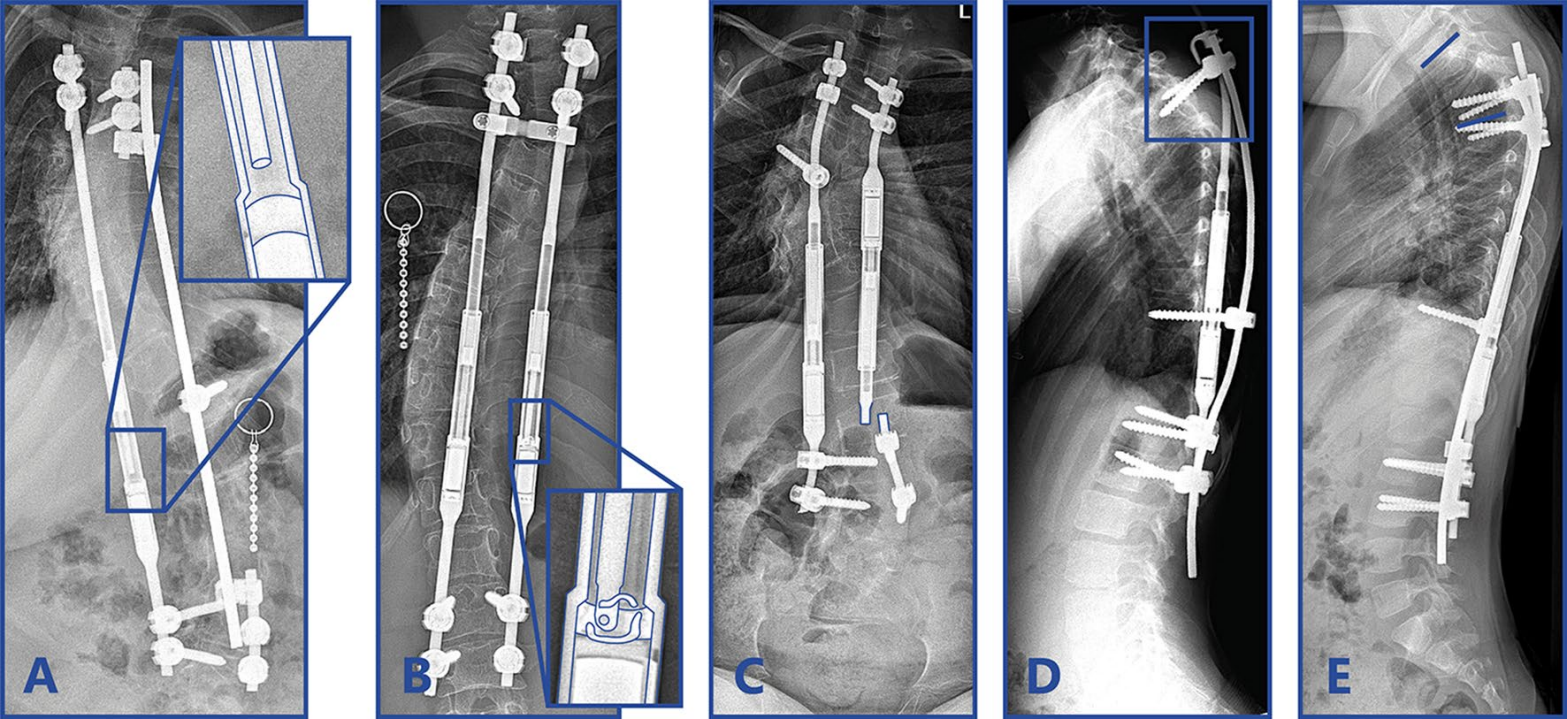
Magnetically controlled growing rod complications. Examples of Magnetically Controlled Growing Rod complications. **a** Actuator rod that is broken and that is disengaged from the rest of the implant. **b** The actuator rod is disengaged from the actuator pin and radial bearing debris is present in the actuator portion of the MCGR. **c** Rod fracture close to the distal foundation after 1.5 years. **d** Anchor failure of the proximal hook and pedicle screws. **e** Proximal junctional kyphosis

**Fig. 4:**
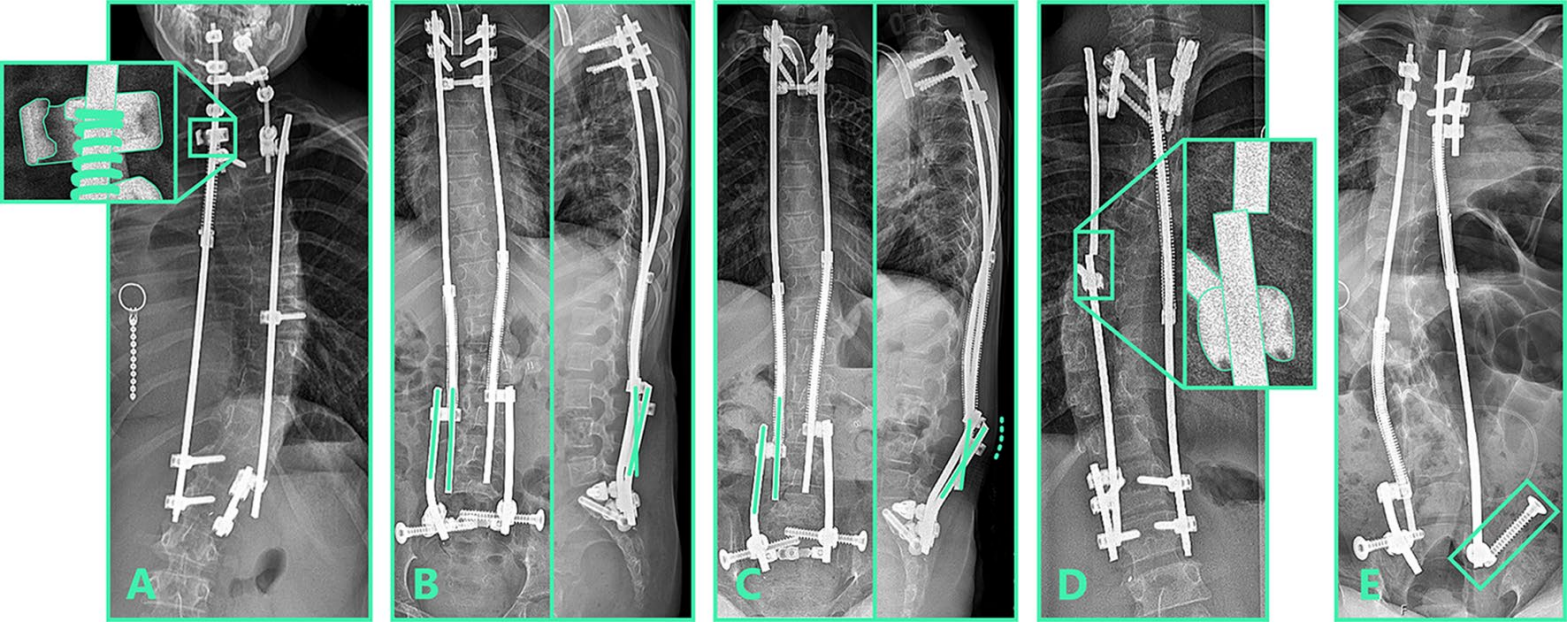
Spring distraction system complications. Examples of spring distraction system complications. **a** Fatigue failure of sliding side-to-side connector. **b** Post-operative radiograph showing the angle that the rods make in the coronal and sagittal plane. **c** After several years of follow-up, distraction caused kyphosis between the sliding and the static rod that resulted in prominence (dashed line). **d** Rod fracture near the apical screw. **e** Distal anchor failure. The iliosacral screw backed out of its original iliosacral trajectory

Complication profile for both groups can be seen in Table 3, a timeline of all complications is reported in Supplement 1. Radiographs of representative complications for both groups can be seen in Fig. 3 (MCGR) and Fig. 4 (SDS). In the (hybrid) MCGR group, the most common complication was failure to distract (8/20 complications), which was diagnosed in these eight patients after a mean of 3.3 ± 1.4 years (range 1.2–6.3). In seven cases of failure to distract, a re-operation was performed. In these cases, the dysfunction of the rods was confirmed during surgery and the MCGRs were explanted and returned to the manufacturer for further analysis. Radiographs taken before re-operation showed a clear failure mode of the MCGR actuator in two patients. Both rods displayed the previously described “crooked-rod sign”, which was followed by a driving pin fracture in one patient (Fig. 3a) and a fracture of the radial bearing (and the driving pin) in the other (Fig. 3b) [17]. The other implant-related complications included four anchor failures, two rod fractures, three cases of PJK, two wound complications and one post-operative neurologic injury, which recovered completely after surgical re-exploration.

In the SDS group, the most frequent complication was implant prominence (5/18 complications), due to increased kyphosis of the rods in the side-to-side connector (Fig. 4b, c). Since the sliding hole in the connector is 1 mm oversized, it is possible for the sliding distraction rod to angulate with the fixed rod due to the off-axial distraction forces. The other complications included three distal iliosacral screw failures, four rod complications, two cases of side-to-side connector failure, three wound complications and one case where the rod grew out of the side-to-side connector. This last patient showed exceptionally fast growth that quickly outpaced the free length of the rod.

### Re-operation rate

Of the 20 complications in the (hybrid) MCGR group, 14 (70%) necessitated a single re-operation. Combining some complications into a single re-operation, and including one re-operation due to reaching the maximum length of the MCGR, 13 re-operations were required in total, corresponding to 0.9 re-operations/patient. Three patients exhibiting PJK and one patient with failure to distract did not require a re-operation. This latter patient did not have much remaining growth left and did not receive a definitive fusion due to increased surgical risk. Of the 18 complications in the SDS group, 16 (89%) necessitated one or more re-operations. Fifteen complications necessitated a single re-operation, one complication necessitated two re-operations. Combining several re-operations, and including two spring re-tensioning re-operations, 17 were required in total, or 0.9 re-operations/patient. One superficial wound dehiscence and one late superficial infection did not require a re-operation. In both groups, no complication required abandoning growth-friendly treatment.

### Survival analysis

**Fig. 5:**
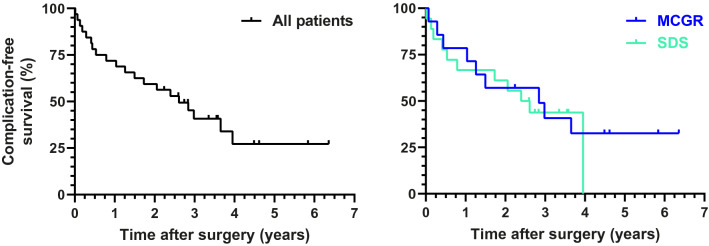
Kaplan–Meier analysis of complication-free survival. Survival time to the occurrence of a complication was evaluated for all patients (left) and for the MCGR and SDS groups separately (right)

Figure 5 shows the Kaplan–Meier survival analysis of all patients combined and of the (hybrid) MCGR and SDS groups separately. Median survival time for all patients was 2.6 years with no significant differences between groups (MCGR 2.8 years; SDS 2.5 years; *p* = 0.673). This indicates that after 2.6 years, half of the patients included in the study had suffered from at least one complication. As the proportional hazard assumption was violated (Fig. 5 shows that the survival functions of the groups cross several times), the hazard ratio between groups was not calculated.

### Implant growth

**Fig. 6:**
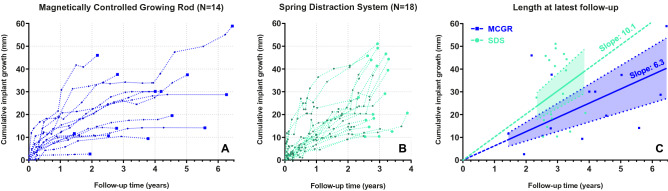
Cumulative implant lengthening over time. Cumulative implant length over time is plotted for the MCGR **a** and the SDS **b** group. The measured values (dashed) of each patient are shown. With the cumulative implant length at latest-follow-up, a linear regression was performed for each group **c**. The intercept-free slopes and their 95% confidence intervals are shown

Cumulative implant growth in the (hybrid) MCGR and SDS patients is shown in Fig. 6a, b. At latest follow-up, 9/18 (50%) of SDS patients showed a cumulative implant growth that exceeded 10 mm/year. For the (hybrid) MCGR group, this occurred in only 2/14 (14%) patients. The cumulative implant growth in both groups was compared in a linear regression analysis shown in Fig. 6c. The linear regression slope of the SDS group equalled 10.1 mm/year (95% CI 7.6–12.7), which was significantly higher (*p* = 0.017) than the MCGR slope of 6.3 mm/year (95% CI 4.2–8.3).

## Discussion

This study investigated complication and implant data from 2 different cohorts. Although this is not the optimal study design, we believe the current single-centre comparison of both implant systems is relevant as it highlights the strengths and weaknesses of both technologies. As also reported by other studies, the implant- or procedure-related complication rate of growth-friendly systems is high, between 0.11 and 0.38/patient/year [5, 18–22]. Normalised for follow-up, complication rate for the (hybrid) MCGR and SDS groups in our study was 0.35 and 0.33 complications/patient/year, respectively.

Failure to distract was the most common (hybrid) MCGR complication, with rods failing to distract in 8/14 patients after a mean of 3.3 years. This obviously impacted the mean implant growth rate (6.3 mm/year), which was significantly lower than the growth observed in the SDS group (10.1 mm/year). The growth provided by the SDS is more in line with what can be expected from physiological spinal growth [23, 24]. Failure to distract is frequently reported in MCGR literature [4, 5, 25]. Mechanical explantation studies attribute this to the extreme frictional forces that the drive mechanism has to withstand [7, 25]. The fact that the actuator portion of the rod cannot be contoured and thus more contouring must take place proximally or distally results in significant off-axis loading, which exacerbates this issue, and which may also be the reason for the high rates of anchor pull-out and proximal junctional kyphosis [18]. These vulnerabilities are inherent to the MCGR design, which is why these complications remain prevalent in the literature, despite several implant iterations [4, 25]. Smaller actuator dimensions could mitigate some of these issues and lower complication rate, although at the expense of a reduced lengthening potential and/or distraction force. In addition, a more dynamic coupling could further decrease internal friction and implant stresses.

In contrast to most other studies investigating the MCGR, we routinely used a hybrid MCGR construct, where a single MCGR is combined with a contralateral sliding rod. Adding the contralateral sliding rod provides apical control and reduces the risk of rod fracture compared to single rod constructs. We have shown previously that hybrid MCGRs provide similar curve correction and spinal growth compared to bilateral constructs [13, 14]. The current study shows that, especially for longer follow-up times, the mechanical failure rate of this hybrid MCGRs is comparable to that of bilateral MCGRs, where mechanical failure is seen in 50–88% of patients during treatment [5, 20–22, 26]. However, as our hybrid strategy differs in several ways from the recommended bilateral MCGR configuration, our results with respect to complication profile and implant growth cannot be extended to bilateral MCGR configurations.

The most frequent complication in the SDS cohort was implant prominence, caused by increased implant kyphosis. This is a direct result of posterior distraction forces combined with a single side-to-side connector that allows for residual bending in the sagittal plane. Currently, we use two stacked side-to-side connectors, which makes this increase of implant kyphosis impossible. However, this has the disadvantage of causing more friction and wear with potential effects on growth, which emphasises that the sliding connection with off-label use of these connectors is suboptimal. In addition, the use of the iliosacral screw initially caused distal anchor complications, these are now prevented with routine use of distal cross-connectors. We believe that with an improved low-friction axial stable bearing and improved iliosacral fixation, the complication rate of the SDS could be reduced further. The effect of such implant changes on curve correction, spinal growth, and incidence of complications will be subject of further investigation.

The SDS provides dynamic loading of the spine, i.e. it allows the implant to transmit load forces to the spine, harnessing the dampening potential of the intervertebral disc. This is in contrast to static implants like TGR and MCGR, where forces are transmitted mostly through the implant. This dynamic loading of the SDS theoretically decreases mechanical stress on the anchors and rods [27]. It may also attenuate stress-shielding that takes place in the segments between the anchors, preventing vertebral osteopenia, which may prove advantageous for final fusion surgery [28–30]. In the current study, the expected reduction in rod stresses did not lead to a decrease in rod fractures in the SDS group (SDS 3/18 patients; MCGR 2/14 patients), although this rate is likely biased due to the application of thinner 4.5 mm rods in the SDS group. Currently, we mainly implant 5.5 mm rods; whether this will prevent rod fractures is subject of investigation as part of a continuous design improvement cycle. Other differences between both groups in the current study include a relatively larger proportion of neuromuscular patients in the SDS group (50% vs 29%), which explains the increased incidence of complications with iliosacral fixation in this group. Fortunately, deep SSIs were uncommon in both groups, likely due to routine usage of intrawound vancomycin powder.

Strengths of the current study includes the fact that the data is obtained from 2 comparable, prospective cohorts, both treated in a single tertiary spine centre. The assessment of procedure-related complications with pre-specified criteria and the use of two observers, make our results repeatable and robust. However, there were several important limitations. First, this study is a retrospective analysis of prospectively collected data, and therefore there is the risk of confounding, selection bias and experience bias. Despite extensive experience with TGR before the (hybrid) MCGR cohort, the team (composed of the same staff during both cohorts) had another 2–3 years more experience with growth-friendly implants at the time of the SDS cohort. In addition, the follow-up for the SDS group is therefore generally shorter. While using follow-up adjusted complications rates mitigates this issue in part, it is possible that certain complications commonly occur within a certain time frame. Depending on whether this window presents early or late following surgery, the complication rate for the SDS group may have been over- or underestimated in this study. Third, patient characteristics and implant configurations were varied and sample size was limited. Finally, these are only intermediate follow-up results. To definitively assess complication rate, patients should be followed at least until final fusion and probably longer [31–33].

## Conclusion

In the (hybrid) MCGR and SDS cohorts, 71% and 61% of patients suffered from at least 1 complication, with a follow-up adjusted complication rate of 0.35 and 0.33 complications/patient/year, respectively. Median complication-free survival across all patients was 2.6 years. There were differences in complication profile between both groups, such as the high rate of failure to distract leading to significantly lower implant growth in (hybrid) MCGR patients, compared to SDS patients (6.3 mm/year vs. 10.1 mm/year). The typical failure mode for the SDS was implant prominence following implant kyphosis. These data may guide future implant improvements of both innovative systems.

## Supplementary Information

Below is the link to the electronic supplementary material.

Supplementary file1 Supplement 1: Complication timeline. The time at which each complication occurred is shown for both MCGR and SDS patients (EPS 5824 KB)

## Declarations

### Regulatory status

At the time of writing, the SDS does not have FDA approval or CE-marking.
